# Comparison of visible imaging, thermography and spectrometry methods to evaluate the effect of *Heterodera schachtii* inoculation on sugar beets

**DOI:** 10.1186/s13007-017-0223-1

**Published:** 2017-09-13

**Authors:** Samuel Joalland, Claudio Screpanti, Frank Liebisch, Hubert Vincent Varella, Alain Gaume, Achim Walter

**Affiliations:** 1 Syngenta Crop Protection Münchwillen AG, Schaffhauserstrasse, 4332 Stein, Switzerland; 2 Institute of Agricultural Sciences, ETH Zürich, Universitätstrasse 2, 8092 Zurich, Switzerland

**Keywords:** Plant phenotyping, Visible imaging, Spectrometry, Thermography, Sugar beet, Nematode, Root, Semi-field

## Abstract

**Background:**

Phenotyping technologies are expected to provide predictive power for a range of applications in plant and crop sciences. Here, we use the disease pressure of Beet Cyst Nematodes (BCN) on sugar beet as an illustrative example to test the specific capabilities of different methods. Strong links between the above and belowground parts of sugar beet plants have made BCN suitable targets for use of non-destructive phenotyping methods. We compared the ability of visible light imaging, thermography and spectrometry to evaluate the effect of BCN on the growth of sugar beet plants.

**Results:**

Two microplot experiments were sown with the nematode susceptible cultivar *Aimanta* and the nematode tolerant cultivar *BlueFox* under semi-field conditions. Visible imaging, thermal imaging and spectrometry were carried out on BCN infested and non-infested plants at different times during the plant development. Effects of a chemical nematicide were also evaluated using the three phenotyping methods. Leaf and beet biomass were measured at harvest. For both susceptible and tolerant cultivar, canopy area extracted from visible images was the earliest nematode stress indicator. Using such canopy area parameter, delay in leaf growth as well as benefit from a chemical nematicide could be detected already 15 days after sowing. Spectrometry was suitable to identify the stress even when the canopy reached full coverage. Thermography could only detect stress on the susceptible cultivar. Spectral Vegetation Indices related to canopy cover (NDVI and MCARI2) and chlorophyll content (CHLG) were correlated with the final yield (R = 0.69 on average for the susceptible cultivar) and the final nematode population in the soil (R = 0.78 on average for the susceptible cultivar).

**Conclusion:**

In this paper we compare the use of visible imaging, thermography and spectrometry over two cultivars and 2 years under outdoor conditions. The three different techniques have their specific strengths in identifying BCN symptoms according to the type of cultivars and the growth stages of the sugar beet plants. Early detection of nematicide benefit and high yield predictability using visible imaging and spectrometry suggests promising applications for agricultural research and precision agriculture.

## Background

The rapid development of sensitive tools for plant phenotyping allows the assessment of very complex traits such as root morphology, biomass, leaf characteristic, yield related traits, biotic and abiotic response [1–3]. In most cases, phenotyping approaches are tested independently under a given scenario which does not facilitate the objective comparison of the methods tested. Often, the different methods are investigated at various scales (field or greenhouse) by following diverse protocols (cultivar, type and level of infestation, growth duration). Sugar beet is an interesting crop since the harvested organ develops vegetatively, thereby integrating environmental effects over time. It has recently been shown that beet development is reflected by aboveground development facilitating the use of shoot phenotyping procedures for yield estimation and disease effects [4]. On sugar beet, limited studies have been published which makes it difficult to evaluate the advantages and disadvantages of the different phenotyping approaches to characterize nematode symptoms.

Nematodes are soil borne parasites that occur naturally in soil. They cause annually up to 20% of yield losses in crops such as soybean, cotton, cereals, tuber crops, legumes, fruit and vegetables [5]. Sugar beet is a root crop which is widely cultivated in Europe and North America for sugar production. The sugar beet cyst nematode *Heterodera schachtii* (Schmidt) is a major threat and can cause severe beet damage and compromise the final yield. It has been demonstrated that there is a strong link between number of nematodes and crop performance such as shoot development and root biomass accumulation [6, 7].

In order to manage the damage caused by nematodes, dedicated strategies have been developed. A first approach consists of evaluating whether the level of infestation in the field is above a given economical threshold thereby justifying specific nematode control methods. However, soil sample analyses are expensive and technically difficult because of the cluster distribution of BCN in the field [8, 9]. Thus, many samples per hectare are required to achieve a reasonable estimation of the potential crop damage.

To reduce costs and increase the spatial resolution of BCN soil pressure evaluation, non-destructive methods have been developed [10]. It is worth noting that BCN occurs in patches in the field, has a low mobility, and causes diverse and rather generic visible aboveground symptoms, for example stunted growth, decreased chlorophyll content and canopy wilting [7, 11]. All this makes BCN an appropriate target for non-destructive phenotyping method development. Several remote sensing methods to detect stress caused by nematodes have already been successfully tested on a variety of crops such as potato, soybean or sugar beet. These methods are mainly based on imaging and non-imaging multi- and hyper spectral measurements, with the calculation of spectral vegetation indices (SVIs) [12–14].

Hillnhütter et al. [15] demonstrated the potential of normalized difference vegetation index (NDVI) to evaluate the symptoms caused by BCN on sugar beet plants under controlled conditions. Use of specific SVIs to predict the final beet yield and the nematode population in the soil has also been reported in field experiments [16]. Schmitz et al. [17] showed the ability of remote sensing thermography at field level to detect small changes in the canopy temperature of BCN-infested sugar beet. Thus, thermography and spectrometry appear to be suitable phenotyping methods for the detection of belowground symptoms caused by BCN. However, these systems require the use of expensive devices and complex data analysis methods.

Alternatively, visible imaging technology can be used for sugar beet phenotyping. Such a technology is cheaper than the aforementioned technologies, since it uses low cost sensors and the devices are easy to handle and calibrate [18]. The projected shoot area of the plants is usually calculated and used as a parameter to predict shoot biomass in different plant species [19–22]. Particularly in sugar beet, the use of visible images showed very promising results in discriminating, at an early plant developmental stage, BCN-infested and non-infested plants in the greenhouse [4]. In this study, the “digital canopy area” parameter calculated was a suitable proxy for shoot and root biomass estimation during the first 2 months of growth.

Beside the need to identify damage caused by nematodes and to evaluate the degree of infestation in the field, the use of phenotyping tools plays a role in agricultural research and development activities aiming at the discovery and development of new solutions for nematode control. In most of the cases, the evaluation studies aimed at evaluating the efficacy of the solutions by looking at the impact on the final yield. This implies that trials need to be kept up to harvest and last 3 months or longer. Using non-destructive measurements to get early insights regarding the efficacy of new solutions (compounds or cultivars) on the yield potential would allow to reduce the duration and costs of the trials, and to increase the testing cycles per year. Overall such new tools can have a substantial impact on the efficiency of compound screening or development of new cultivars.

The present study compares the ability of several traits (canopy area, canopy temperature and SVIs) obtained with three different phenotyping devices (visible imaging, thermography and spectrometry visible) to identify and characterize stress caused by BCN on sugar beet plants at the semi-field level. More specifically, the main objectives were to:

(i) Compare the ability of the three phenotyping methods to detect stress generated by BCN on nematode susceptible and tolerant sugar beet plants,
(ii) Evaluate the capability of the methods to predict sugar beet yield,
(iii) Evaluate the potential of visible imaging to detect benefit of a contact nematicide.

## Methods

### Plant cultivation

**Fig. 1:**
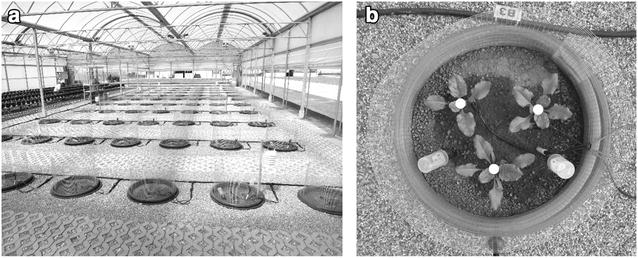
**a** Overview of the semi-field platform with 70 microplots. **b** Top view of a microplot 416 °Cd. White dots represent the three “sowing locations”

Studies were conducted in 2014 and 2015 on a polytunnel area located in the Syngenta Research Centre in Stein (Switzerland). The area was equipped with a microplot system (Fig. 1a), which simulates real field conditions and allows to monitor the main environmental conditions. The experimental layout includes 70 microplots consisting of a pot in pot system. One 150 L plastic container (65 cm diameter and 60 cm depth) is nested inside of another, with both recessed in the ground up to the rim to reduce fluctuation of soil temperature.

The nematode susceptible cultivar *Aimanta* (Syngenta AG, Basel, Switzerland) and the nematode tolerant cultivar *Bluefox* (Syngenta AG, Basel, Switzerland) were used in 2014 and 2015 trials, respectively. The soil used was non-sterile sandy loam (56% sand, 31% silt, 11% clay, pH 7.7, 2% O.M.). A commercial seed treatment consisting of Thiram, Hymexazol, Thiamethoxame and Tefluthrine (6 + 14 + 60 + 8 g_ai_/unit (one sugar beet unit is 100,000 seeds)) was applied to the seeds to avoid early insect attack and fungal disease. Six seeds were sown per microplot at three different locations (two seeds per location) (Fig. 1b). Two weeks after sowing, three seedlings were left in each microplot (one seedling kept per location) simulating a sowing density similar to the real sowing density adopted under field conditions (100,000 seeds per hectare).

### Preparation of the soil and nematode inoculum

Cysts of *H. schachtii* were cultured at the Syngenta research centre in Stein. Cysts were coming from greenhouse oilseed rape plants cultured in loess soil. Infested soil was prepared by mixing the sandy loam soil with the amount of infested loess soil to reach a final level of 600 eggs and juveniles (J2) per 100 cm^3^ soil. Only the upper layer (corresponding to a volume of 40 L) of each microplot was infested.

In the 2015 experiment, an additional treatment including soil infested with cysts of *H. schachtii* + *Fosthiazate* nematicide (ISK Bioscience Corporation, Concord, OH, USA) was added [23]. *Fosthiazate* was applied as granules of Nemathorin 10G product (Syngenta AG, Basel, Switzerland) at the same time as the soil infestation with a final rate of 30 kg/ha. A randomized complete block design with ten replicates was used in the 2 years. Experimental settings and main crop management operations are reported in Table 1.

**Table 1:** Summary of the experimental settings and the crop management operations during the two microplot experiments

	2014	2015
Sugar beet Cultivar	Nematode susceptible *Aimanta*	Nematode tolerant *Bluefox*
Nematode infestation level	600 eggs and J2 per 100 cm^3^ of soil	600 eggs and J2 per 100 cm^3^ of soil
Treatments	(1) Non-infested (2) Nematode infested	(1) Non-infested (2) Nematode infested (3) *Fosthiazate* treatment
Sowing	May 6th 2014	May 4th 2015
Fertilizer application (Osmocote^®^ granules)	–	440 °Cd
Insecticide application	534, 1094 °Cd	857, 1419 °Cd
Fungicide application	1830 °Cd	2012 °Cd
Harvest	September 10th (2200 °Cd—127 das)	August 27th (2190 °Cd—115 das)

°Cd represents the thermal time

**Fig. 2:**
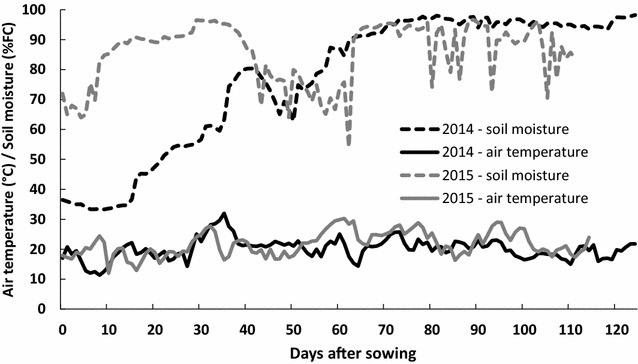
Evolution of daily average air temperature and soil moisture during 2014 and 2015 trials. Soil moisture is expressed as percentage of the field capacity (FC). The end of the lines corresponds to the respective harvest dates

In both years, microplots were equipped with sensors to monitor air temperature (www.msr.ch) and soil moisture (www.plant-care.ch). Soil sensor technology is based on the microthermic measurements of soil moisture. Details of the environmental conditions are shown in Fig. 2. Thermal Time (TT) expressed in degree days (°Cd) was calculated using trait temperature as: TT = Σ *if* ≥ 0 $\left({\frac{{T_{\max} + T_{\min}}}{2}} \right) - T_{base}$, with T_base_ of 1.1 °C [24].

In 2015, soil moisture during the first 40 days was higher than in 2014. In both years, soil moisture conditions were sufficient to allow a homogenous growth of the seedlings. It is worth noting that high air temperatures were observed during the sugar beet emergence (between 6 and 9 days after sowing) in 2015 (21 °C on average).

### Evaluation of plants and nematodes

After harvest, fresh weight of beets was determined for each plant. Dry weight of the leaves was measured after a drying period of 72 h (70 °C). Final nematode population was assessed by sampling 1000 g of soil. Soil sampling was performed between five and 20 cm depth in the middle of each pot. All the soil samples were subsequently sent to an external lab (ClearDetection, Wageningen, NL) for analysis of the number of cysts and number of eggs and larvae per 100 cm^3^ of dry soil according to EPPO method 1/25 (http://pp1.eppo.int/). Plant growth stages (GS) were defined according to the BBCH scale [25].

### Visible imaging

**Fig. 3:**
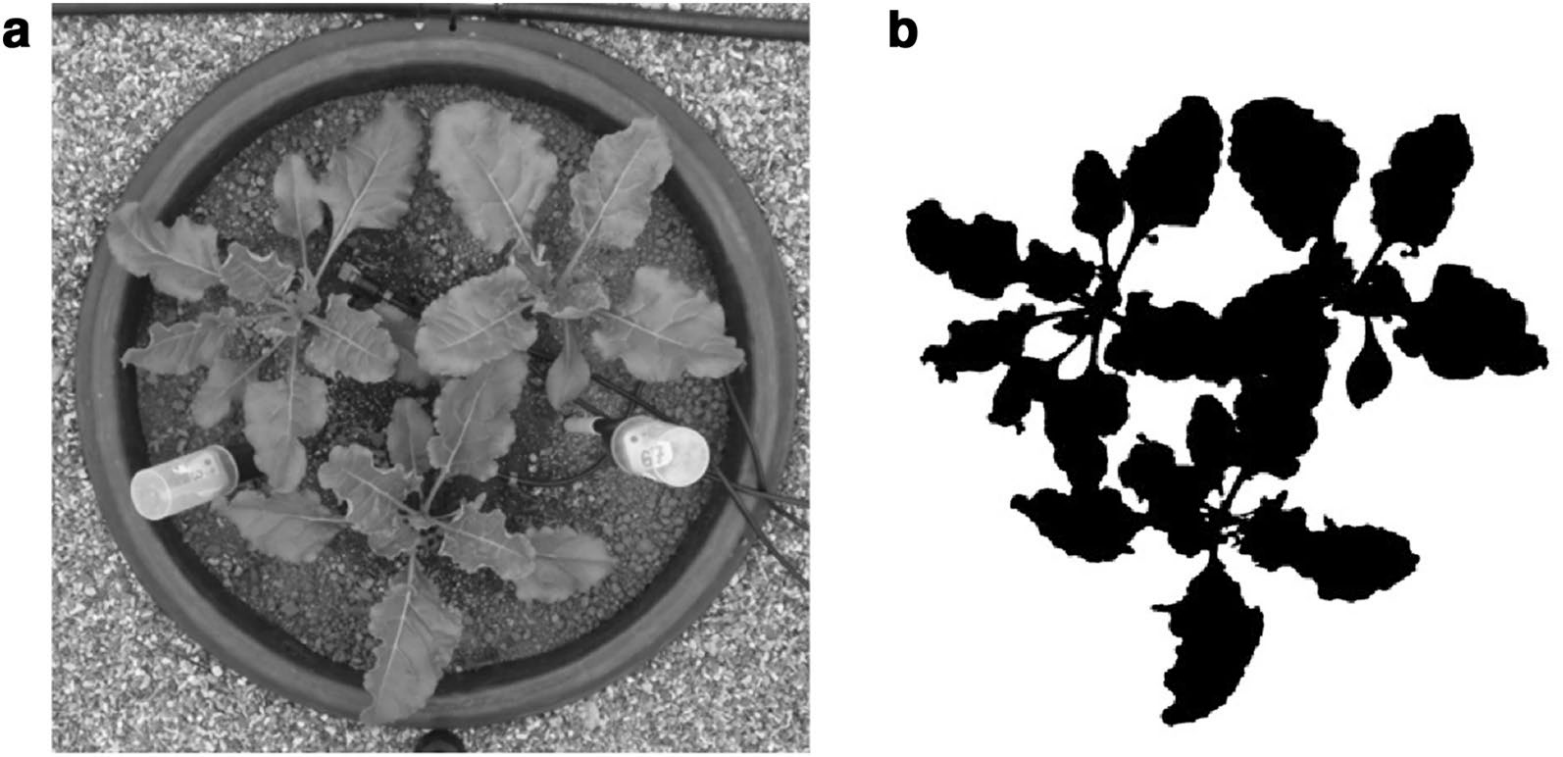
**a** Raw visible image taken from the top. **b** Image after processing 534 °Cd (susceptible cultivar *Aimanta* in 2014)

Canopy visible images were captured from seedling emergence up to 1300 °Cd every 2 or 3 days using a digital camera Canon S100 (Canon, Tokyo, Japan). The device was mounted on a mobile monopod and images were obtained from 1.8 m above plant canopy with a resolution of 0.0029 cm^2^ pixel^−1^. The monopod was held vertically in order to have the camera centred in the middle of the pot. To optimize image processing, photos were captured, when possible, under cloudy conditions early in the morning using the automatic settings of the camera. Fifteen minutes were necessary to acquire the 70 images which prevented any changes in the illumination and therefore also the necessity for white balancing. Raw pictures (Fig. 3a) were processed using *ImageJ*, the Java-based open-source image processing and analysis program (https://imagej.nih.gov/ij/), following a workflow described by Joalland et al. [4] based on an image segmentation proposed by Woebbecke et al. [26]. This fast and non-invasive method allows to evaluate the “digital canopy area” (green area) at different times (Fig. 3b).

### Thermography

**Fig. 4:**
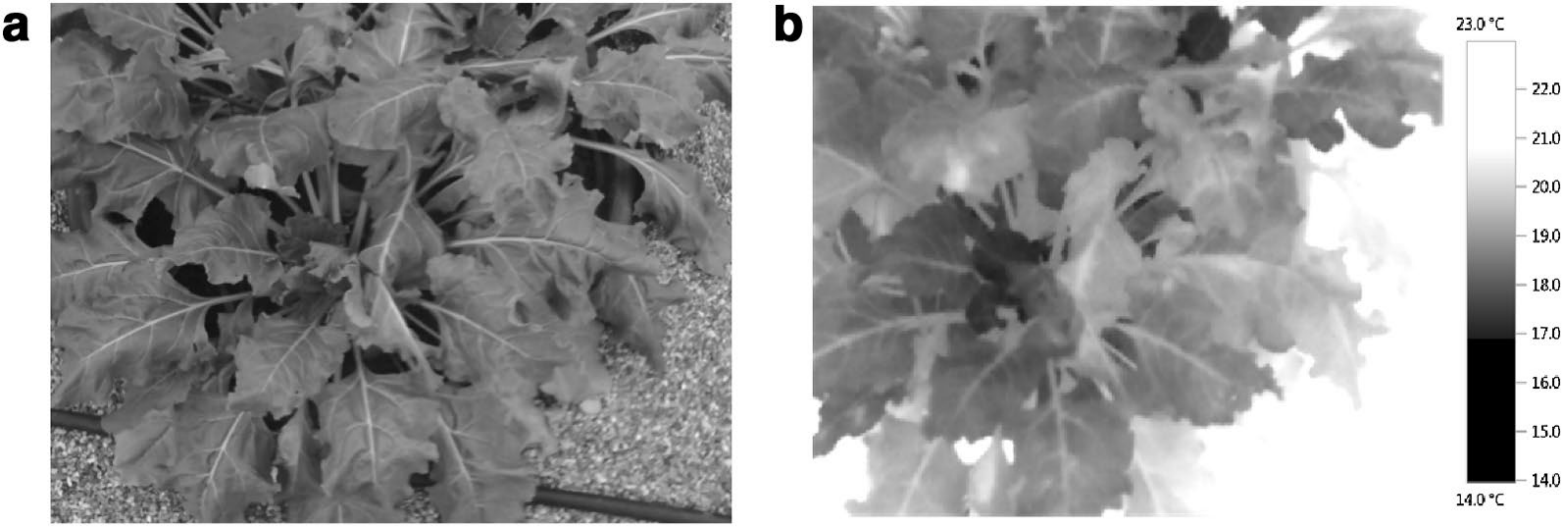
**a** Visible and **b** thermal images obtained simultaneously with the thermal camera 856 °Cd in 2015

Thermal images were acquired using an infrared camera (Testo 885, Testo Ltd, UK). The thermal device was calibrated prior to taking pictures by setting up the emissivity to 96% and the reflected temperature compensation parameter to the current air temperature [27]. Pictures were then taken from the top of each pot in manual mode (autofocus off). Two images were automatically generated by the camera during the image acquisition; one thermal image, in which pixels correspond to temperature value, and one visible image (Fig. 4a, b). A macro was specifically built on *ImageJ* to extract the canopy temperature by combining both thermal and visible images.

For each date and timing of measurement, the Vapour Pressure Deficit (VPD) was calculated according to the equation of Anderson [28] using air temperature and relative humidity. VPD reflects the ability of the air to hold water and it reflects the transpirational demand.

### Spectrometry

#### Data acquisition

Spectral measurements were performed several times during plant development (Table 2) with a non-imaging spectroradiometer (ASD FieldSpec^®^ 4, Analytic Spectral Devices, Boulder, CO, USA) with a spectral range of 350–2500 nm. Spectra were acquired from the top of the microplots at a height of 1.20 m with a 25° field of view using a mobile dark box specifically designed for the microplot experiments. A 120 W halogen lamp (spotlight 120 W, Kent, Lyon, France) was used to provide constant optimum illumination of the canopy inside the box during the measurements. The dark box and halogen lamp combination made the conditions of measurement consistent between microplots and between days.

**Table 2:** Summary of phenotyping measurements during the two studies

	2014		2015	
	Data points #		Data points #	
Visible imaging	33	Emergence to 1300 °Cd	33	Emergence to 1300 °Cd
Thermal imaging	3	935, 1446, 1485 °Cd	5	581, 599, 736, 856, 1347 °Cd
Spectrometry	1	1371 °Cd	6	404, 460, 599, 736, 978, 1618 °Cd

°Cd represents the thermal time

Instrument optimization and reflectance calibration were performed using a Zenith Polymer^®^ (SphereOptics, Germany) 99% reflectance target as white reference before the sample acquisition. Each sample scan represented an average of five reflectance spectra.

#### Spectral vegetation indices

For each date of measurement and each microplot, a selection of 123 published SVIs was computed to reduce the data dimension. SVIs were calculated using ratios of several bands at different ranges of the spectrum. For each measurement date, a correlation matrix was built for the 123 SVIs using control non-infested and control infested treatments. Indices highly inter-correlated to each other (Pearson’s correlation coefficient R > 0.8) were grouped resulting in 20 groups. One SVI was then selected by group which resulted in a final selection of 20 SVIs per date. For the final study, eight SVIs were selected out of 20 following a discriminant analysis between non-infested and nematode infested treatments to reflect the broad range of traits for which the SVIs were initially developed (Table 3).

**Table 3:** Selected SVIs, their respective equations, the aimed detection trait and references

SVIs	Equation	Traits	References
NDVI	(R_800_ − R_680_)/(R_800_ + R_680_)	Biomass, coverage	[29]
MCARI2	(1.5[2.5 (R_800_ − R_670_) − 1.3 (R_800_ + R_550_)])/sqrt((2*R_800_ + 1)^2 − (6*R_800_ − 5*sqrtR_670_) − 0.5))	LAI, coverage	[30]
780/700	R_780_/R_700_	Nitrogen content	[31]
TGI	−0.5[(W_670_ − W_480_)(R_670_ − R_550_) − (W_670_ − W_550_)(R_670_ − R_480_)]	Chlorophyll content	[32]
CHLG	(R_760_ − R_800_)/(R_540_ − R_560_)	Chlorophyll content	[33]
PRI	(R_531_ − R_570_)/(R_531_ + R_570_)	Stress	[34]
NDWI1650	(R_840_ − R_1650_)/(R_840_ + R_1650_)	Plant water status	[35]
HI	(R_534_ − R_698_)/(R_534_ + R_698_) − R_704_/2	Plant health	[1]

### Statistical data analysis

The program *R* [36] was used for analysis of the biological data. Beet fresh weight, leaf dry weight and canopy area of BCN infested and non-infested plants were tested for homogeneity of variance. They were then exposed to analysis of variance (ANOVA) at a probability level of 0.05 using the factor “nematode infestation”. Linear regression models were used to quantify the relationship between final beet fresh weight, nematode population and several phenotyping parameters. Regarding spectrometry data, a discriminant analysis was performed to identify, for each date of measurement, indices that allow to discriminate between control infested and non-infested treatments.

## Results

### Plant fresh weight and nematode population

In both experiments, an artificial inoculation corresponding to 600 eggs and J2 per 100 cm^3^ of soil led to a moderate pressure similar to what can be expected in field situations. Such nematode pressure significantly affected the final beet fresh weight (Table 4). In 2014, beet biomass of nematode infested treatment was reduced by 32% compared to the non-infested treatment for the susceptible cultivar *Aimanta*, whereas in 2015, the final beet biomass reduction was 11% for the tolerant cultivar. Final average leaf dry biomass of infested plants (39.4 g plant^−1^) was significantly lower than that of non-infested plants (47 g plant^−1^) for the susceptible cultivar (−16%) (p < 0.05). This was not the case for the tolerant cultivar where no effect of BCN could be observed on the final leaf dry biomass.

**Table 4:** Effect of BCN on final beet fresh weight and leaf dry weight of sugar beet plants

	Beet fresh weight (g)	Leaf dry weight (g)	Final nematode population (eggs/larvae per 100 cm^3^ soil)
2014—susceptible cultivar *Aimanta*			
Non-infested (control)	1286.1 ± 30.0a	47.0 ± 1.5a	16.7 ± 14.3a
Nematode infested	886 ± 49.0b	39.4 ± 2.2b	13,535.0 ± 1552.0b
2015—tolerant cultivar *Bluefox*			
Non-infested (control)	1230.7 ± 34.2a	58.7 ± 1.2a	50.0 ± 43.4a
Nematode infested	1096.2 ± 42.3b	60.0 ± 2.0a	6950.0 ± 1236.2b

As expected, almost no nematodes were found in the soil of non-infested treatments for both trials. Presence of negligible numbers of eggs and larvae can be explained by the non-sterile field soil that was used for these experiments. A larger number of nematodes was found in the infested pots in 2014 (on average 13,535 eggs and larvae per 100 cm^3^ soil) compared to 2015 (6950 eggs/larvae on average). The average Pf (Final nematode population)/Pi (Initial nematode population) ratio was 27 in 2014 and 11.5 in 2015.

### Non-infested and infested treatments are displayed for 2014 and 2015 experiments

**Fig. 5:**
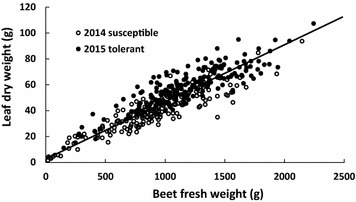
Final leaf dry weight as a function of the final beet fresh weight (n = 381, R^2^ = 0.79, p < 0.01)

For both susceptible and tolerant cultivars, final aboveground biomass was strongly correlated with the belowground biomass (Fig. 5). Linear regression in 2014 and 2015 resulted in R^2^ of 0.82 and 0.74 respectively suggesting that leaf biomass is a good indicator of the beet biomass. The close relationship between above and belowground sugar beet biomass confirms the interesting use of non-destructive phenotyping tools to evaluate the status of the plant canopy over time. By measuring the canopy, the growth of the beet can be indirectly investigated.

### Early stress detection using visible imaging

**Fig. 6:**
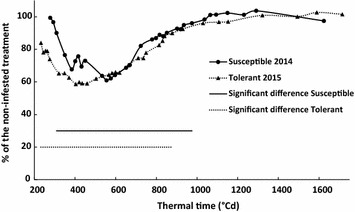
Evolution of the canopy area of infested susceptible and tolerant cultivars. Canopy area is expressed as a percentage of the non-infested treatment. Time periods where the difference in canopy area between infested and non-infested treatments are significant are represented on the figure (p < 0.05)

Evolution of the canopy area of infested sugar beets presented similar patterns for susceptible and tolerant cultivars (Fig. 6). Canopy areas of both varieties were strongly affected by BCN during the first 600 °Cd. From 600 to 1000 °Cd, canopy area differences between infested and non-infested plants decreased due to a combination of leaf overlapping and plant recovering. After 1000 °Cd, differences between infested and non-infested treatments were not visible anymore using the canopy area parameter.

Canopy area allowed the detection of the nematode stress that was applied and the statistically significant discrimination between infested and non-infested plants from 230 °Cd (GS 13) to 880 °Cd (GS 33) for the nematode tolerant cultivar *BlueFox* and from 335 °Cd (GS 14) to 995 °Cd (GS 35) for the susceptible cultivar *Aimanta* (p < 0.05) (Fig. 6). This difference in the timing of the stress detection between the 2 years of trial was most likely caused by the low air temperatures during the first 2 weeks after sowing in 2014 (Fig. 2). This led to a slow and non-homogenous crop establishment and a low nematode pressure. The high variability in canopy area was reflected by the higher coefficient of variation observed (38% in 2014 and 18% in 2015). In summary, the visible imaging method was very sensitive in detecting the damaging effect of nematodes on aboveground plant growth already in very early stages (after the development of the third leaf 335 °Cd).

### Canopy temperature evaluation using thermography

In 2014, canopy temperature differed significantly between treatments throughout the whole observed period (Table 5). Always, canopy temperature of nematode infested plants was significantly higher than the canopy temperature of the non-infested plants. At 1446 °Cd, average canopy temperature of the non-infested treatment was 18.6 versus 19.5 °C for the infested treatment. For the susceptible cultivar, differences between the two treatments increase with increasing VPD.

**Table 5:** Canopy temperature (°C) of non-infested and nematode infested treatments at different times during the season

Susceptible 2014	935 °Cd (57 das)	1446 °Cd (84 das)	1485 °Cd (86 das)
(a)			
Date—Time	02/07—10:30	29/07—15:30	31/07—14:30
Vapour pressure deficit (kPa)	0.37	0.77	1.93
Non-infested	13.7 ± 0.3a	18.6 ± 0.5a	22.8 ± 0.4a
Nematode infested	14.6 ± 0.2b	19.5 ± 0.3b	27.4 ± 0.6b

Tolerant 2015	581 °Cd (36 das)	599 °Cd (37 das)	736 °Cd (44 das)	856 °Cd (52 das)	1347°Cd (74 das)
(b)					
Date—Time	09/06—17:30	10/06—16:30	17/06—17:00	25/06—14:30	17/07—16:30
Vapour pressure deficit (kPa)	0.68	0.87	2.01	2.44	4.88
Non-infested	15.1 ± 0.1a	17.7 ± 0.22a	23.7 ± 0.46a	23.3 ± 0.69a	32.8 ± 0.79a
Nematode infested	15.4 ± 0.09b	18.1 ± 0.18a	23.8 ± 0.41a	24.5 ± 0.43a	33.4 ± 0.75a

(a) Susceptible cultivar in 2014. (b) Tolerant cultivar in 2015. Displayed, are the mean ± standard error of each treatment. Different letters within each column indicate significant differences

In 2015, for the five dates of measurement, the canopy temperature of infested plants was on average 0.5 °C higher than the canopy temperature of the non-infested ones. However the difference was statistically significant only at 581 °Cd. There was no obvious correlation between the differences in canopy temperature of the two treatments and the VPD for the tolerant cultivar.

It can be stated that such canopy temperature differences observed between treatments are caused by nematode stress and not by the environmental condition variability on the platform. In fact, the randomized complete block design of the experiment was set up according to an air temperature gradient which prevented any effect of air temperature variability on the canopy temperature comparison between nematode infested and non-infested treatments. Soil moisture was similar for all the pots at each measurement date.

### Nematode stress identification by a spectrometry approach

In Table 6, indices are grouped according to their relevance in assessing plant biomass, chlorophyll content, water status and general stress. Among the different indices, those related to the biomass, chlorophyll and general stress resulted in better detection of the nematode infestation and damage at the different stages of the crop development. In 2015, from 404 to 736 °Cd (GS 15 to GS 31), SVIs mainly related to plant biomass such as NDVI or leaf area such as MCARI2 were significantly affected by nematodes which confirmed the previous observation concerning the canopy area. At more advanced stages (GS 31 to GS 39), differences could be detected on both susceptible and tolerant cultivars using the CHLG and TGI respectively. The Health Index (HI), which was developed specifically for sugar beet, was particularly effective consistently across the 2 years of investigation and at different stages of crop development.

**Table 6:** Selection of SVIs that allowed to statistically discriminate non-infested and nematode infested treatments

Thermal time (°Cd)	Growth stage	Biomass	Chlorophyll	Water	Stress
2014					
1371	37	NDVI, 780/700	CHLG	NDWI1650	HI
2015					
404	15	NDVI, 780/700, MCARI2	PRI	–	HI
460	16	MCARI2, 780/700	PRI	–	HI
599	20	MCARI2	CHLG, PRI	–	HI
736	31	MCARI2	TGI	NDWI1650	HI
978	35	780/700	TGI	–	–
1618	39	–	PRI	–	–

A comparison of means has been performed (*t* test for independent samples) and the significant SVIs are displayed in the table. SVIs in bold have a p value lower than 5% and the others between 5 and 10%. SVIs were grouped according to the trait they are related to

In the last measurement (1618 °Cd), sugar beet plants displayed additional symptoms of general stresses with early leaf senescence which affected the identification of sole nematode effects.

### Phenotyping parameters and final data

Most of the SVIs in Table 6 were significantly correlated with beet fresh weight and final nematode population in the soil. Correlation coefficients were always higher for the susceptible cultivar compared to the nematode tolerant cultivar (Table 7). On average NDVI, MCARI2 and CHLG were highly correlated with the beet fresh weight (R = 0.69) and the final nematode population (R = 0.78) for the susceptible cultivar. Cumulative canopy area which reflects the ability of the plants to absorb light over the season was significantly correlated with the final beet fresh weight in 2015 (R = 0.54, p < 0.1). In 2014, the weak correlation observed was not significant (R = 0.32). There was no significant correlation between cumulative canopy area and final BCN population in the soil. Canopy temperatures did not show any significant correlations with the final beet fresh weight and BCN population.

**Table 7:** Pearson’s correlation (R) between phenotyping variables at different dates and the fresh weight of the beet and final nematode population in the soil (n = 20, p < 0.1)

Thermal time (°Cd)	Detection trait	Beet fresh weight	Final number of eggs/larvae per 100 cm^3^ of soil
2014—susceptible			
935, 1446, 1485	Canopy temperature	–	–
–	Cumulative canopy area	0.32	–
1371	CHLG	0.80*	−0.79*
	NDVI	0.59*	−0.76*
	MCARI2	0.67*	−0.78*
2015—tolerant			
581, 599, 736, 856, 1347	Canopy temperature	–	–
–	Cumulative canopy area	0.54*	–
404	HI	0.40*	−0.60*
460	780/700	0.37*	−0.71*
599	CHLG	0.41*	−0.42*
	PRI	0.32	−0.61*
736	TGI	0.36	−0.51*
978	HI	0.37*	–

Cumulative canopy area: Integral of the canopy area from sowing until the date when the plateau was reached (1300 °Cd in 2014 and 1100 °Cd in 2015). * indicates significant correlations (p < 0.1)

### Practical application of visible imaging for nematicide research

**Fig. 7:**
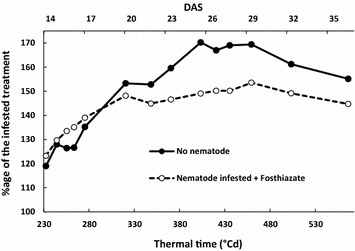
Canopy area of the non-infested and *fosthiazate* treatments as a percentage of the nematode infested treatment (n = 10). Only the first 35 days of growth are represented. From 244 to 560 °Cd both non-infested and *fosthiazate* treatments showed statistically significant higher canopy area than the nematode infested treatment (p < 0.05)

**Fig. 8:**
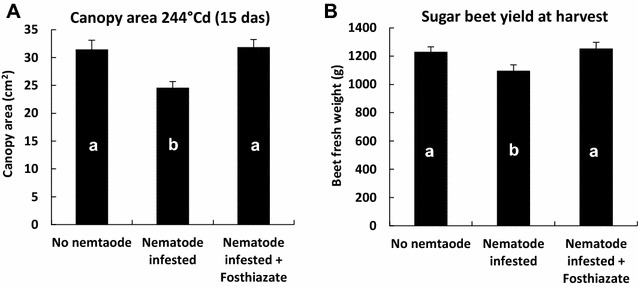
**A** Average canopy area and **B** final beet fresh weight of three treatments from 2015 trial (n = 10). Bars represent the standard error of the mean. Different letters indicate significant differences (p < 0.05, n = 10)

In 2015, clear differences were observed in the canopy area between treatments during the first 35 days of plant development (Fig. 7). 244 °Cd (15 das), canopy area of *fosthiazate* treated plants was 29% higher than canopy area of the nematode infested treatment. At this date, average canopy areas of non-infested and *fosthiazate* treatments were statistically larger than the canopy area of nematode infested treatment (Fig. 8A). Evolution of canopy areas of non-infested and *fosthiazate* treated plants showed similar pattern. Both treatments showed significantly higher canopy area compared to the nematode-infested treatment from 244 to 560 °Cd (GS 16) (Fig. 7).

The same trend was observed in the final sugar beet yield. *Fosthiazate* treatment showed significant benefit in the final beet fresh weight compared to the nematode infested treatment (+14%) (Fig. 8B). Early canopy area differences reflected the final sugar beet yield.

## Discussion

The present study compares three phenotyping techniques in the same experimental settings across 2 years and two sugar beet cultivars. A multi-sensor approach was found to be of advantage for continuous crop phenotyping or monitoring in experimental and field settings [2, 37]. While more studies have been conducted under controlled greenhouse conditions [4, 15], this work was carried out outdoor by simulating conditions that are close to the real field situations comprising soil type, nematode infestation, plant density, duration of the crop cycle, plant canopy and root development. To the best of our knowledge, such a comparison of three phenotyping methods under outdoor conditions to characterize the sugar beet growth has not been published before.

During the 2 years of experimentation, the artificial nematode infestation successfully led to a yield reduction of 32% for the susceptible cultivar and 11% for the tolerant cultivar. These results are consistent with reports from other field and microplot studies [7, 38]. Thus the microplot settings used in this study were successful to simulate a realistic timing of nematode infestations and crop damage. BCN multiplication was 2.5 times higher for the susceptible cultivar compared to the tolerant one. This order of magnitude is consistent with respect to the definition of “nematode tolerance” given by Trudgill [39]. The fact that no differences could be observed in the final shoot biomass for the tolerant cultivar can be explained by the ability of the nematode tolerant cultivar to endure nematode damage and recover during the second part of the growing season [40, 41].

Visible imaging, thermography and spectrometry measurements enabled detection in a non-invasive, dynamic and objective manner of the effect of nematode infestation on sugar beet plants. Digital canopy area extracted from top-view visible images is a suitable tool to evaluate the effective plot-based canopy area. This parameter is taking account of different morphological components of the sugar beet such as the number of leaves, the area of the leaves and the plant architecture [42]. Canopy area appeared particularly suitable to dynamically characterize the early growth of the sugar beet plant from sowing to an advanced vegetative stage (GS 35). In a previous study carried out under greenhouse conditions, the “digital canopy area” parameter was identified as a proxy to estimate the shoot and root biomass of the sugar beets [4]. Such dynamic prediction of leaf biomass using visible images was also reported and used for high throughput phenotyping on other crops under greenhouse conditions [43, 44]. In the present study we demonstrated that the digital canopy area can be adapted to, and is effective in, outdoor conditions by looking at clusters of plants simulating the natural seed density expected in real field conditions. Overall, the top down visible imaging method showed its strength in the early evaluation of the degree of growth inhibition of the plant biomass. The nematode tolerant cultivar did not prevent BCN affecting the early plant development. Surprisingly, in the early growth stages, the canopy area reduction was higher for the tolerant cultivar compared to the susceptible cultivar which indicates that the tolerance mechanism does not prevent early nematode damage [45]. Benefits in very early plant growth (244 °Cd) observed with the use of fosthiazate showed the ability of a contact nematicide to protects the root by suppressing the first generation of J2 hatching from the cysts and to ensure yield benefit compared to the untreated plants [46].

Canopy temperature reflects plant water status, stomatal conductance and transpiration rate of the leaves [27, 47, 48]. It has been shown that nematodes strongly decrease water uptake of the roots which increases the stomatal resistance and consequently reduces the leaf evapotranspiration [49, 50]. In 2014, significantly higher canopy temperatures were observed for the nematode infested sugar beets compared to the non-infested plants. These results are consistent with previous observations made by Schmitz et al. [17] where a correlation between canopy temperature and nematode density was observed. Temperature difference between the two treatments increased with VPD. Infested susceptible plants had difficulties in cooling down their leaves when air conditions become constraining (VPD > 1.5). Most likely, infested plants were not able to keep up the high transpiration rate because of nematode damage at root level which compromised water uptake. The tolerant cultivar *Bluefox* behaved differently. Tolerance mechanisms allow sugar beet plants to maintain their transpiration rate even under high VPD.

Spectrometry measurements allowed the calculation of SVIs that reflected specific agronomical or physiological traits such as chlorophyll content, water content, biomass or photosynthesis rate [51–54]. The present study showed that specific SVIs allowed to differentiate between nematode infested and non-infested plants. Nematodes have an effect on different physiological parameters in both susceptible and tolerant cultivars. On the tolerant cultivar, most symptoms occur during the first 2 months of growth whereas on the susceptible cultivar, symptoms persist at more advanced growth stages since the plants are not able to recover from the infestation. The 2015 experiment helped to associate a type of BCN stress with the growth stages or time period where it occurs. The performance of indices related to the biomass and chlorophyll content was variable depending on the growth stages and among them MCARI2, 780/700 and TGI were the most promising. A close relationship has been demonstrated between the value of TGI index and the leaf chlorophyll content on a variety of crops [32, 55]. Such effect of BCN decreasing the leaf chlorophyll content was also reported by Schmitz et al. [11]. Nematode effect on the leaf water content was low in the tolerant cultivar which confirmed the limited effect of nematodes (also observed with thermography) in reducing transpiration rate on a tolerant cultivar. Two SVIs appeared suitable from early growth stages (GS 15) to advanced stages (GS 39) in detecting the stress caused by nematodes; HI and PRI. Health index (HI) uses two spectral regions centered on 700 nm and 534 nm. Reflectance near 700 nm is a feature of green vegetation and chlorophyll content whereas reflectance around 534 nm is an indicator of photosynthetic function [34, 52]. Thus, HI can be classified as a general stress index [1]. Photochemical reflectance index (PRI), based on reflectance at 531 and 570 nm, reflects the light use efficiency [56]. Although HI and PRI are not nematode specific, they appear suitable in detecting nematode stress over the whole season under semi-field conditions.

Correlations between SVIs and final sugar beet biomass demonstrated the ability of spectrometry in predicting final yield on both susceptible and tolerant cultivars. In particular, CHLG and MCARI2 were the best SVIs to predict final yield on susceptible cultivars. Close relationship between beet fresh weight and nematode incidence make the correlation between SVIs and BCN populations evident. Correlations were higher for the susceptible cultivar compared to the tolerant cultivar because of the larger range of beet fresh weight that was observed. Cumulative canopy area was also correlated with the final sugar beet yield suggesting a close relationship between the early plant growth and the final yield of the sugar beet. Such relationship between early phenotyping parameters and final yield is not so clear with other crops such as maize or wheat where the early plant growth does not always reflect the final plant yield as reported by Tekrony and Egli [57], Egli and Rucker [58] and Sankaran et al. [59]. In this respect, sugar beet appears a suitable crop for early yield prediction using phenotyping measurements.

Our results obtained with visible phenotyping showed that a slight delay in plant growth during the first 30 days had a significant effect on the final yield. Similar results were highlighted in a previous field study by sowing seeds of sugar beets at different timings to simulate a delay in the plant development [60]. The larger the canopy, the greater is the use of incident radiation. Olthof [61] observed higher damage on the plant when seeds were sown directly in nematode infested soil than when the infestation occurred 2 weeks after sowing. Early growth delay observed for the nematode infested plants could not be compensated during later growth of the crop. Thus, it appears crucial to avoid stress during the first growth stages of the sugar beet [62]. The *fosthiazate* effect in 2015 supports this point. In this experiment, *fosthiazate* nematicide was used as additional “positive control”. This nematicide acts by suppressing nematode hatching from the cysts and paralyzing juveniles and it is known to provide a strong root protection during the first month of the plant growth [46]. According to the rate that was applied (30 kg ha^−1^) and the concentration required for biological activity, it is likely that the *fosthiazate* effect in the soil stopped after 6–8 weeks [46, 63]. However, the early protection enabled a good development of the seedlings and insured yield benefit compared to the nematode infested plants. This result is of interest for crop protection research. Under moderate nematode pressure, protection of sugar beet plants against nematode damage should occur from sowing to 1200 °Cd (GS 25). Late nematode infestation did not significantly affect the plant growth of tolerant cultivars.

Given the complicated nature of the investigated nematode-plant interaction with belowground damage and unspecific symptoms displayed in the canopy, the phenotyping techniques evaluated here provided very encouraging results with potential applications in the area of sugar beet research. It can be stated that, visible imaging, thermography and spectrometry compared in the present investigation are complementary tools and are particularly suitable for automation. Field phenotyping platforms with multiple sensor systems will therefore be a valuable tool to improve crop performance via optimized management schedules [64, 65].

## Conclusions

In conclusion, the study demonstrated that it was possible to use non-invasive and non-destructive technologies to characterize the dynamic of the plant growth and detect stress symptoms caused by BCN on nematode susceptible and tolerant sugar beets. While thermography only showed the ability to detect BCN stress on a susceptible cultivar, spectrometry and visible imaging technologies allowed the indirect observation of BCN damage on both susceptible and nematode tolerant cultivars and to give a prediction of the yield potential. In addition, the three different techniques have their specific strength at different points in time reflecting particular growth stages of the sugar beet. Visible imaging was the earliest stress indicator whereas spectrometry and thermography could identify the stress still when the canopy reached full coverage. Further applications of these tools could be developed for controlled environment and field situations. Under control conditions, canopy area has a great potential to be used as an early parameter to predict the degree of inhibition of the plant biomass caused by BCN and to quantify the degree of benefit from a new compound. Under field conditions visible image analysis, alone, may not be sufficiently specific to identify nematode damage because canopy area reduction can be caused by other types of stress. Therefore this technique would need to be combined with other approaches (e.g. spectrometry; thermography and/or soil sampling).

## Abbreviations

BCN: beet cyst nematode
das: days after sowing
GS: growth stage
SVIs: spectral vegetation indices
